# Deep Drawing of High-Strength Tailored Blanks by Using Tailored Tools

**DOI:** 10.3390/ma9020077

**Published:** 2016-01-27

**Authors:** Thomas Mennecart, Hamad ul Hassan, Alper Güner, Noomane Ben Khalifa, Mohamad Hosseini

**Affiliations:** Institute of Forming Technology and Lightweight Construction, TU Dortmund University, Dortmund 44227, Germany; Hamad.Hassan@iul.tu-dortmund.de (H.H.); Alper.Guener@iul.tu-dortmund.de (A.G.); Nooman.Ben_Khalifa@iul.tu-dortmund.de (N.B.K.); Mohamad.Hosseini@rub.de (M.H.)

**Keywords:** tailored blanks, hybrid deep drawing tools, high strength steels, tailored tools

## Abstract

In most forming processes based on tailored blanks, the tool material remains the same as that of sheet metal blanks without tailored properties. A novel concept of lightweight construction for deep drawing tools is presented in this work to improve the forming behavior of tailored blanks. The investigations presented here deal with the forming of tailored blanks of dissimilar strengths using tailored dies made of two different materials. In the area of the steel blank with higher strength, typical tool steel is used. In the area of the low-strength steel, a hybrid tool made out of a polymer and a fiber-reinforced surface replaces the steel half. Cylindrical cups of DP600/HX300LAD are formed and analyzed regarding their formability. The use of two different halves of tool materials shows improved blank thickness distribution, weld-line movement and pressure distribution compared to the use of two steel halves. An improvement in strain distribution is also observed by the inclusion of springs in the polymer side of tools, which is implemented to control the material flow in the die. Furthermore, a reduction in tool weight of approximately 75**%** can be achieved by using this technique. An accurate finite element modeling strategy is developed to analyze the problem numerically and is verified experimentally for the cylindrical cup. This strategy is then applied to investigate the thickness distribution and weld-line movement for a complex geometry, and its transferability is validated. The inclusion of springs in the hybrid tool leads to better material flow, which results in reduction of weld-line movement by around 60%, leading to more uniform thickness distribution.

## 1. Introduction

Due to the increase in demand of different strengths in structures, several types of tailored blanks like tailor-rolled or tailor-welded exists as mentioned in Merklein *et al.* [1]. The possibility to select different sheets of different thickness, strength, and material properties enables the designer to distribute the material optimally. This leads to lighter structures, higher strengths, and joining before forming, which lowers the production costs [2]. The use of tailor-welded blanks (TWBs) has been introduced in beginning of the 1980’s by Audi [3] in Germany and Toyota [4] in Japan. The main benefits of these blanks exist in the possibility of weight reduction and in the capability to reduce joining processes in the press shop. Mostly, due to the weld line on the surface and shifts of thickness, tailored blanks are used as structural parts in the body-in-white of cars for improving crash performance. Typical applications are pillars (A and B) or the inner structure of car doors, where different strengths in different regions are required. 

In case of forming tailored blanks consisting of sheets with different thickness, the forming tools have to be designed with such a shift and the weld-line movement has to be taken into account to avoid failures such as wrinkles or cracks [5]. Weld-line movement occurs especially when forming tailored blanks with high differences in the stress strain behavior and with a high thickness ratio [6] that facilitate the movement of the weld line. The investigation of the stretch-forming behavior of tailored blanks made out of dissimilar material combinations using dual-phase (DP) steels are carried out by Panda *et al.* [7]. It was concluded that, due to the non-uniform strain distribution, the weaker high-strength low-alloy (HSLA) sheet metal failed close to the weld, resulting in a decrease in the limiting dome height of tailored blanks. Weld-line movement in deep drawing of cylindrical cups has been investigated, and the typical failures like cracks and wrinkles are pointed out when using materials with different thickness [8,9]. The weld-line movement and the formability in general are described analytically by Kinsey and Cao [9]. Several investigations exist on the improvement of the formability of tailor-welded blanks and the reduction of weld-line movement. An application of draw bead in the die is proposed by Heo *et al.* [10], who added restraining force to control the flow of thinner material during deformation, resulting in a significant reduction in weld-line movement. However, the higher restraining force results in early thinning on the thinner side and subsequently leads to the failure of the tailored blanks during flange drawing. The control and adjustment of the blank holder force can lead to a minimization of the weld-line movement [11]. The die cushion of the press is replaced by a nitrogen cylinder system consisting of six nitrogen cylinders. The system thus behaves like a multipoint pressure control system capable of adjusting the blank holder force around the periphery of the sheet. Kinsey and Cao showed the possibility of a reduction of weld-line movement by clamping the blank locally with the use of hydraulic pressure in a segmented tool [12,13]. Variable blank holder pressure is also suggested by Kinsey and Wu [6] to control the movement of the weld line.

The use of segmented tools for forming tailored blanks enables the application of different blank holder forces on the sheets. Although usually segmented, the tool materials do not differ and allow withstanding high loads. It means that, in one region, the lifetime of the tool is overestimated. Three benefits mentioned for the use of light tools are the reduction of cycle time, lower energy consumption, and handling during the installation of the tool in the press [14]. A major benefit of the servo press technology is that the ram can be stopped and accelerated without reaching the dead center, and the cycle time can therefore be reduced [15]. By reducing the weight of the tools, the effectiveness of the servo presses can be increased. To improve the forming behavior of tailored blanks, to decrease the weld-line movement, and to make the process more energy efficient, the aim of this work is to replace the tool material of one segment by using hybrid tools as described in the studies of Witulski *et al.* [16]. With the use of such tools, the forming of parts with DP600 material is possible, and the lifetime of such tools can reach a number of 1000 parts without the appearance of failures on the tool or on the formed sheet [17]. Another known quantity-optimized tooling technology is the use of nickel-shell-based tools [18]. This is a hybrid tool system that consists of a wear-resistant active tool surface (nickel) and a polymeric base. This strategy has a potential of cost savings of up to 40% over conventional tool technologies. Mennecart *et al.* [19] pointed out that the use of polymer (polyurethane) and hybrid tools (polyurethane strengthened by fiber reinforcement) has some other advantages too, such as the homogenization of pressure distribution or the capability to include elements to control the local blank holder pressure. As described in the studies of Endelt, Tommerup and Danckert [20,21], the use of media to apply a load in different regions can improve the forming results of high-strength steels. 

The scope of this work covers the use of segmented tools in deep drawing by replacing a steel tool segment by one made out of polymer and fiber reinforcement to improve the formability of tailored blanks. Investigations are performed to show the influence of these tools on the formability of TWBs made of two different high-strength steels. Tailored blanks made of DP600 and HX300LAD are deep drawn with six different tool configurations. The polymer-based hybrid tools are produced as described by Kolbe [17]. In order to analyze the formability of the material, numerical simulations are also performed for a simple cup geometry. The strain and thickness distributions are analyzed to investigate the effect of different parameters on formability. A validation of the simulations for cup geometry is also carried out by the experiments. In order to show a possible application of such tools in press shops, simulations are also performed with different setups for a complex geometry. 

## 2. Materials and Methods 

**Figure 1:**
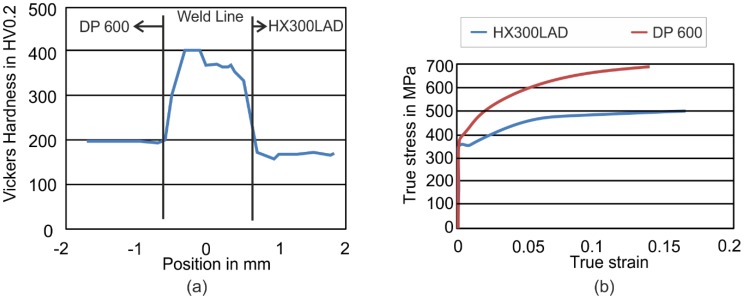
(**a**) Hardness distribution across the weld line for Tailor Welded Blank; (**b**) flow curves of DP600 and HX300LAD steels.

The tailored blanks used in these investigations consist of a micro-alloyed steel HX300LAD and a dual-phase steel DP600. The sheets were welded by a CO_2_ Laser with a power of 3.6 kW by ThyssenKrupp Steel Europe AG (Essen, Germany). The width of the weld line was approximately 1.0 mm with a heat-affected zone of a maximum of 100 μm. Hardness measurements across the weld line are shown in the Figure 1a. While the hardness in the weld line was about 360 HV0.2 and 400 HV0.2 near the DP600, the hardness values of the steels were nearly half of the weld line’s level (DP600: 200 HV0.2, HX300LAD: 160 HV0.2). The blank thickness of both materials was 1.0 mm.

The flow curves of both materials are presented in Figure 1b. To consider the anisotropy, Lankford’s parameters were measured for the directions 0°, 45° and 90° with respect to the rolling direction (Table 1). 

**Table 1:** Lankford’s parameters due to anisotropic behavior.

Steel Grade	*r*_0_	*r*_45_	*r*_90_
HX300LAD	1.08	0.98	1.32
DP600	0.86	0.90	1.08

In the scope of this work, a cylindrical cup with a drawing depth of 20 mm and a diameter of 60 mm was deep drawn. The diameter of the initial sheet was 110 mm. The die entrance radius was 10 mm and the punch radius was 5 mm. The punch speed was 150 mm/s. The blank holder force remained constant at a level of 25 kN. In the deep drawing process, the sheets were lubricated with oil with a dynamic viscosity of 168 mm^2^/s at 40 °C. 

In these investigations, utilized punch and blank holder were one-piece steel tools, and the dies were casted with different polymers with the methodology described by Witulski *et al.* [16]. Three polymers namely GM725-7 (K1), GM 725-7 (K2) fiber-reinforced (aramid) and GM 986-1 (K3) having different Young’s modulus were used to investigate the influence of tool elasticity on the formability of tailored blanks. The aramid layers were stacked three times in a rotation of 30° each so that isotropic behavior could be reached. Between each cured layer, a layer of 1 mm of polymer was cast to smoothen peaks or irregularities. The polymer K1 had a higher density than the other two polymers after the solidification. Polymer K2 had a higher Young’s modulus than the other two polymers due to the inclusion of fiber reinforcement. The roughness had a high influence on the restraining force and on the material flow in the forming process. Due to high loads, the asperities on the surface deformed elastically and plastically, leading to an increase of the contact area. Therefore, with increasing normal loads on polymer (polyurethane) tools, the friction force also increased [22]. The different tool materials with their mechanical properties are listed below in Table 2. 

**Table 2:** Material properties of different tool materials used in investigations [17].

Material	Density ρ (t/mm³)	Young’s Modulus *E* (MPa)	Poisson Ratio ν	Roughness Average *R*_a_ (µm)	Roughness Peak *R*_z_ (µm)
Tool Steel	7.89 × 10^−9^	2.1 × 10^5^	0.3	0.1	0.9
K1: GM725-7	1.75 × 10^−9^	8.0 × 10^3^	0.4	1.7	9.8
K2: GM 725-7 Fiber-reinforced (aramid)	1.67 × 10^−9^	14 × 10^3^	0.3	1.5	8.1
K3: GM 986-1	1.15 × 10^−9^	7.9 × 10^2^	0.3	1.6	7.9

**Figure 2:**
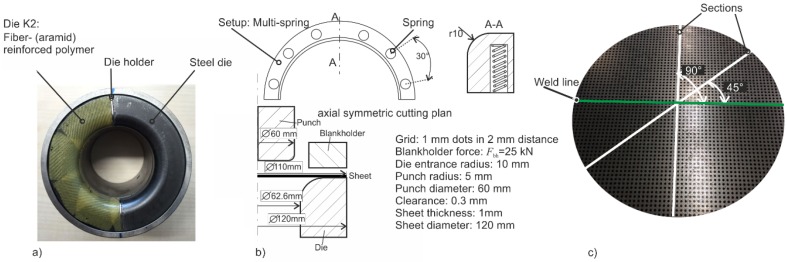
(**a**) Segmented die setup with steel and reinforced polymer type K2; (**b**) spring-reinforced die setups and axial symmetric cutting plan; (**c**) screen-printed grid on initial tailored blank with sections in two orientations related to the weld line.

With the polyurethane K3 (GM 986-1), two further configurations with springs were generated. These springs acted as local stiffness element and increased the stiffness of polymer locally. This also compensated the softer behavior of K3 polymer as compared to the others. The polymer dies were strengthened with metallic springs with a diameter of 6 mm and a maximum force of 0.25 kN that acted as locally added stiffness elements. In one configuration, only one spring was inserted in the polymer in the middle of the die-half (90°). The remaining wall thickness in the die was 3 mm. In the second configuration, six springs were inserted at equal distance of 30° in the die-half. The position of the springs is shown in Figure 2b.

With these materials six variants of forming dies were produced:

- Setup 1: Steel /Steel
- Setup 2: Steel /K1
- Setup 3: Steel /K2 (shown in Figure 2a))
- Setup 4: Steel /K3
- Setup 5: Steel /K3 Single-spring
- Setup 6: Steel /K3 Multi-springs

The two halves were inserted in a die holder and fixed from the external side by screws such that the clearance of 0.3 mm remained constant during the deep drawing process and the dies did not move relative to each other. In the forming experiments, the weld line of the initial sheet was positioned longitudinal to the contact area of the two halves. When using the polymer tools, the softer material (HX300LAD) is always formed by the polymer die. Due to weld-line movements caused by different stress-strain behavior and anisotropy, it is possible that the blank may have moved, and the materials may have changed their contact partner. After forming, the weld-line movement was analyzed optically by measuring with a caliper. 

In order to measure the strains, a grid of dots with a diameter of 1.0 mm and a distance of 2.0 mm was printed on the initial sheet with the technique of screen printing as shown in Figure 2c. After forming, the cups were analyzed by optical measurement system ARGUS (GOMmbH, Braunschweig, Germany) for the strain measurement. Here, the sections in 90° and 45° to the weld line were taken into account (see Figure 2c). With the system ATOS (GOMmbH), the digitalization of the 3D Shape was performed. The tactile sheet thickness was measured by the use of a caliper in determined sections (90° and 45° to the weld line) for the validation purpose.

**Figure 3:**
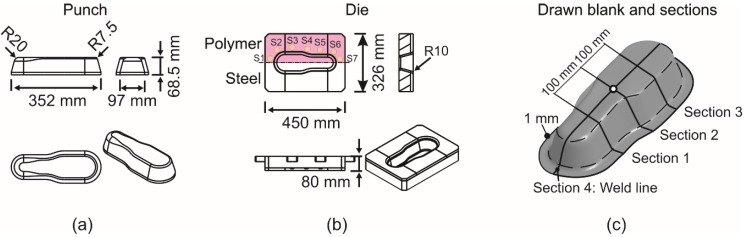
Dimensions of tool for complex geometry in mm. (**a**) Punch; (**b**) die; (**c**) drawn sheet and sections.

The numerical investigations in this work were performed for the deep drawing of a cup and a complex geometry with LS-DYNA (LSTC, Livermore, CA, USA) using explicit time integration scheme. Sheets were modeled after a mesh convergence analysis with 1 mm of element size. Fully integrated shell elements (type 16) were used for the simulation of deep drawing with seven integration points over the thickness. The important dimensions of these geometries are shown in Figure 2 and Figure 3, respectively. Both sheet materials were modeled using Hill’48 model, and the flow curves were extrapolated by the Swift rule. The weld line was modelled as a line, and its mechanical properties were neglected due to a small width of 1.0 mm and a relatively higher strength of the weld zone as suggested by Panda *et al.* [7]. The common nodes on the weld line of both materials were merged together. For this reason, the translational and rotational degrees of freedom of the nodes from the two base materials were coupled. In this simulation model, the punch and blank holder were modeled as rigid bodies. Dies were modeled as elastically deformable part with solid elements to simulate the effects of tool deformation and the inclusion of springs. Springs were modeled as the node sets on the die surface, and an extra load was applied in the form of pressure to simulate their effect on the material flow. For the cup geometry, the total CPU time reached a value of 354 min (4 CPU on Intel i7). No mass and time scaling were used. The time step used was 6.9 × 10^−9^ s. The springs were located 5 mm away from the die inflow radius. The locations of springs for cup geometry and complex geometry (springs marked as S1 to S7) are shown in Figure 2b and Figure 3b, respectively. The thickness and strain distributions were analyzed on specific sections shown in Figure 2c and Figure 3c, respectively.

## 3. Results and Discussion

### 3.1. Geometry Analysis of Experiments

**Figure 4:**
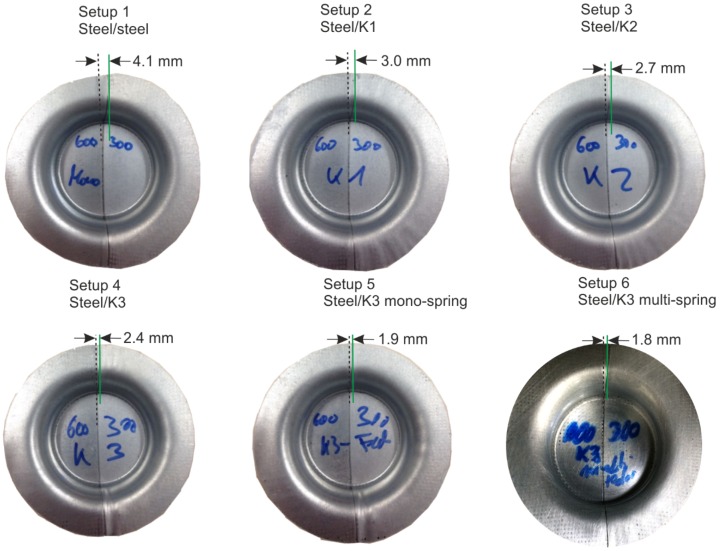
Formed cups in different die-setups with distance of weld-line movement (left side of TWB: DP600, right side: HX300LAD).

**Figure 5:**
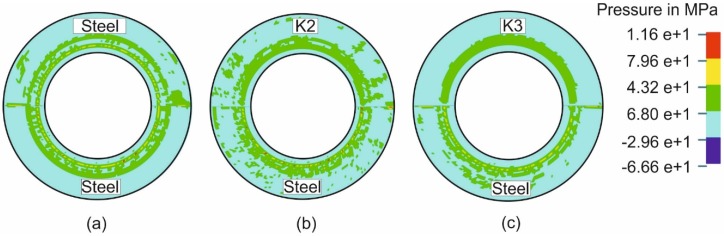
Pressure distributions in Setup 1 (**a**) and Setup 3 (**b**) and Setup 4 (**c**).

In the forming experiments, which were conducted four times per setup, the influence of the different dies on the weld-line movement of the formed blanks was visible (Figure 4). These different weld-line movements, which were reproducible, can be explained in four different aspects:

1. Due to the higher flow stress of the DP600 steel, the softer steel HX300LAD started to flow earlier, which means that the weld line moved towards the DP600 material. This effect can be especially seen when the forming was carried out with the two steel halves. For the experiment with Setup 1 (Steel/Steel), the movement is about 4.1 mm.
2. When forming was done with polymer tools, the pressure distribution became more homogenous on the HX300LAD blank (Figure 5), which led to a uniform holding of the flange. It caused a reduction of movement of about 1.1 mm in the case of Setup 2 (from 4.1 mm in Setup 1 to 3.0 mm in Setup 2) and 2.3 mm (from 4.1 mm in Setup 1 to 1.8 mm in Setup 6) in the case of the setup with the springs (Setup 6).
3. Due to the fact that the real blank thickness of the steels varied between 0.98 mm and 1.0 mm, the elastic behavior of the polymer tools was able to compensate slightly for this kind of variation. For this reason, the blank holder force acted on both grades of steel more homogenously and led to a reduction in weld-line movement by the application of polymer die.
4. If high load is applied on a deformable surface (like polymer), the asperities at the surface of the polymer tool are crushed and the surface becomes smoother. In this case, asperities of the harder steel sheet penetrated into the smoother surface of the polymer die. With increasing normal force, the effect of this surface interaction was more pronounced, and, as a result, the friction coefficient increased, which led to a higher restraining force.

With more elastic die materials, the movement of the weld line was able to decrease. Although this forming behavior could be improved successfully, the deviation in the die entrance becomes higher with the elastic tools.

Figure 5 shows the reason of the minimization of weld-line movement due to elastic deformation of the die and especially the die entrance in comparison with Setup 1 (steel/steel) (Figure 5a). Figure 5b,c show the pressure distribution in the middle of the punch travel for the tool for Setups 3 and 4. Here, Setup 4 (steel/polymer K3) (Figure 4c) shows higher pressure and a more homogeneous pressure distribution in the die entrance than Setups 1 and 3 (Figure 5b). This can be explained by the different contact angles in this area due to the deformation. When the zone of contact was larger, the restraining force was caused by friction increases.

**Figure 6:**
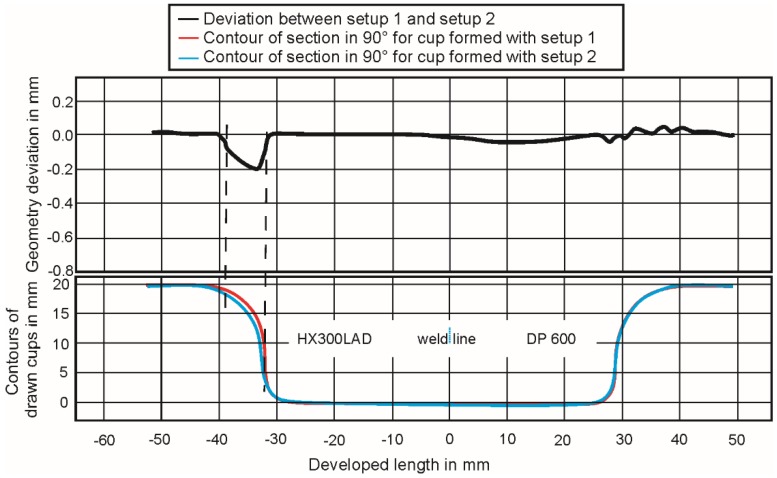
Geometry deviation due to the use of elastic dies.

Figure 6 shows the deviation of geometries when forming with Setups 1 and 2. With Setup 2, the maximum deviation of −0.20 mm occurred. Although this deviation is not high (1 mm thick sheet), it could be due to the elastic behavior of the polymer die in the region of HX300LAD. For the polymer K3, the deviation on the die entrance radius was about −0.16 mm. A possible reason could be that, in the production of these tools, air bubbles cannot be avoided in 100%, and this can lead to a little loss of stiffness under load. The material properties in Table 2 are representative for ideal casting processes without air cavities or bubbles. Due to the appearance of small wrinkles in the formed parts with using Setups 2 and 3 upon further analysis, the focus is on Setups 4 and 6. 

**Figure 7:**
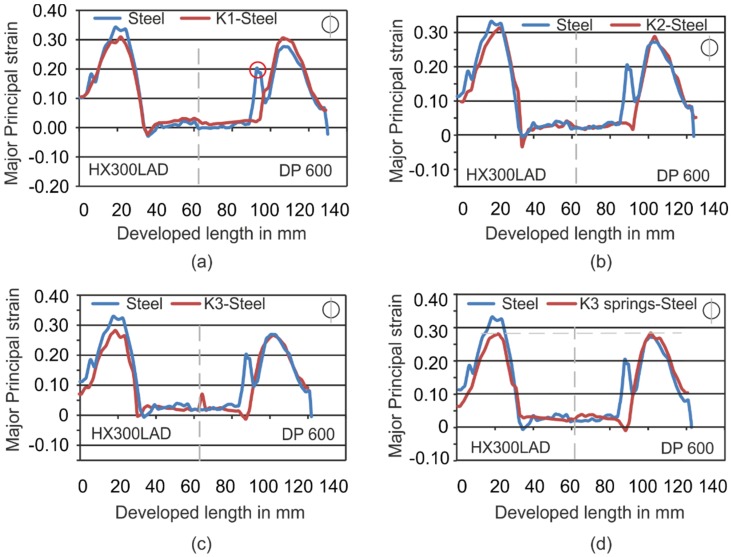
Comparison of major strains of Setup 1 with Setup 2 (**a**); Setup 3 (**b**); Setup 4 (**c**) and Setup 6 (**d**).

In Figure 7, the experimental results of different setups are compared to Setup 1. Here, differences in the strain evolution can be recognized. While major strains of 0.3 are reached with Setup 1, Setup 2 and Setup 3 (Figure 7a,b), the maximum strains on the side of DP600 and HX300LAD were able to be reduced in Setup 4 and Setup 6 (Figure 7c,d). This also led to a reduction of thinning, which can be seen in Figure 7. The homogenization of the strain can be explained by the homogenization of pressure, as also described by Kolbe [17]. In Figure 7d, it can also be seen that the strain distribution was further improved by the inclusion of springs in the die. In contrast to the Figure 7c, an increase in the strain can be seen for HX300LAD. The springs in the die are responsible for the increase in the stretching in the sheet while at the same time keeping the maximum strain to a lower level, as compared to the steel die. It is for this reason that the weld-line movement is reduced quite effectively in Setup 6. The peaks (as one marked with a red circle in Figure 7a) in the strain distributions on the side of DP600 appear due to the damage of the measurement grid in the area where the punch radius made contact. The same phenomena can also be recognized for DP600 in Figure 9b and Figure 10c.

**Figure 8:**
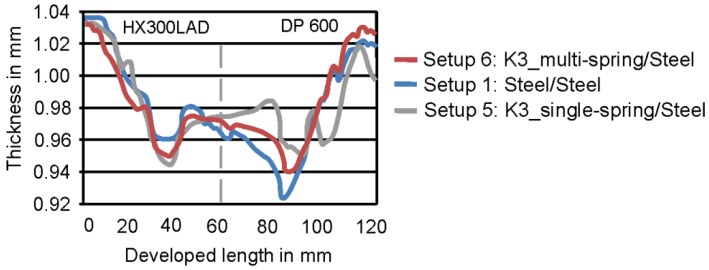
Comparison of thickness distribution for setups 1, 5 and 6.

Figure 8 shows the thickness of the tailored blanks for the section in 90° for Setups 1 (steel/steel), 5 (steel/spring) and 6 (steel/multi-spring). It can be seen that there is a possibility for thickness reduction or homogenization of thickness distribution when using elastic tools like in Setup 5 and Setup 6. In Setup 1, it can be seen that the thickness reduction of HX300LAD was lower, as compared to DP600. By using a single spring in the die (Setup 5), the thickness reduction could also be influenced; however, due to anisotropy, this distribution was not uniform. By the application of multiple springs (Setup 5), however, the distribution was made even better. Although the DP600 has lower Lankford parameters, and the flow of this material is more out of the thickness, the minimum thickness was able to be increased from 0.92 mm to 0.96 mm for Setup 5 and to 0.94 for Setup 6.

With the application of different tool setups, different forming behaviors were achieved. From the experimentally drawn parts of the cup geometry, small wrinkles could be seen for Setup 2 (Polymer K1/steel) and Setup 3 (Polymer K2/steel). The parts formed with Setup 4 (Polymer K3/steel), Setup 5 (steel/spring), and Setup 6 (steel/multi-spring) were deep drawn without failure. These three setups were based on Polymer K3. The thickness distribution was more homogenous using Setup 6 (Figure 8). For this reason, the numerical analysis was performed for Setups 1, 4 and 6.

### 3.2. Numerical Analysis

#### 3.2.1. Cylindrical Cup Geometry

**Figure 9:**
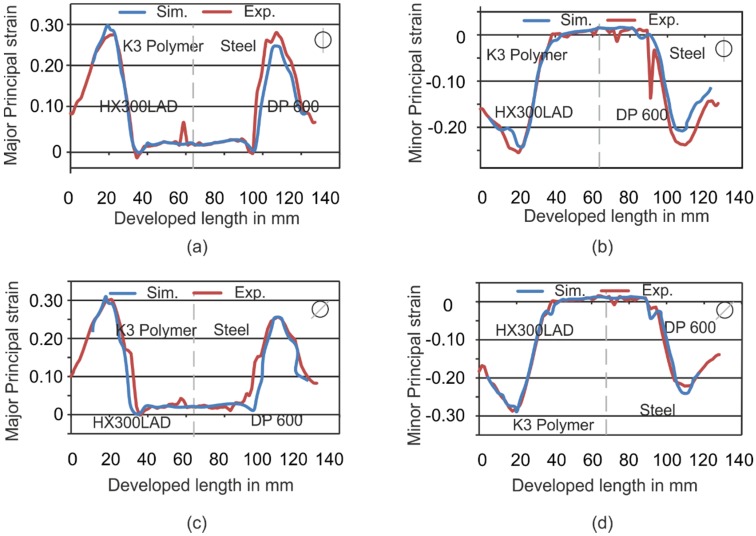
Comparison of major and minor strains of simulations and experiments for Setup 4. (**a**) Major strain for section in 90°; (**b**) minor strain for section in 90°; (**c**) major strain for section in 45° and (**d**) minor strain for section in 45°.

A strategy for the accurate finite element modeling was developed to analyze the problem numerically. This strategy was applied to investigate the strain and thickness distribution and validated experimentally for the cup geometry. After the validation of numerical simulations for the cup geometry, the numerical analysis for a complex geometry was performed to check the transferability of the strategy. For the validation of the cup geometry, two selected sections (Figure 2c) were considered. Figure 9 shows the comparison of strains for both sections for Setup 4. In Figure 9a,b, the strains in section at 90° are analyzed. In Figure 9c,d, the major and minor strains in 45° are plotted. In Figure 9a, a maximum major strain deviation of about 0.02 on the side of K3 die and a deviation of 0.01 at the side of the steel die can be seen. In Figure 9b, the minor strain on the side of the polymer die fits very well. In Figure 9c, there is a difference in strain of more than 0.1, which is very high. This may be due to defects in the die as a result of the casting process. That can be a strong reason for the deviation between the experiments and the simulation. 

**Figure 10:**
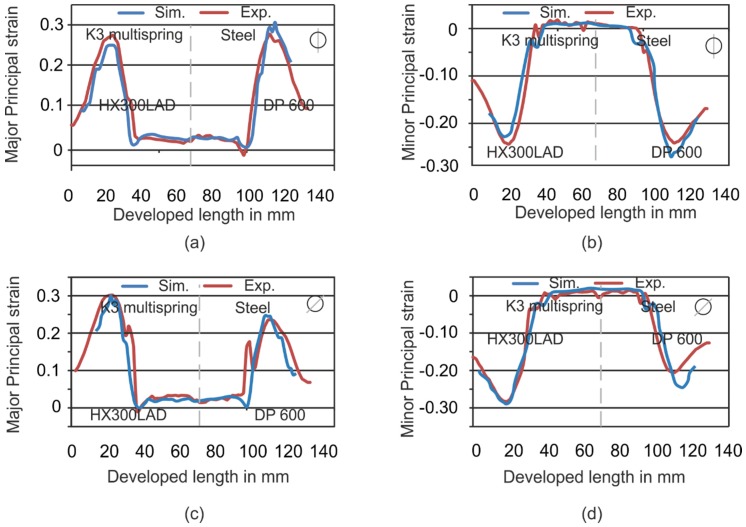
Comparison of major and minor strains of simulations and experiments for Setup 6. (**a**) Major strain for section in 90°; (**b**) minor strain for section in 90°; (**c**) major strain for section in 45° and (**d**) minor strain for section in 45°.

In Figure 10, the major and minor strains were also compared for Setup 6 for both directions. The experiments can be simulated with high accuracy and very low deviations (in Figure 10a–c) for this setup, although, in Figure 10d, a deviation of approximately 0.03 in the minor strain exists. With these comparisons, it could be stated that the simulations can predict the forming behavior in different setups. 

#### 3.2.2. Complex Geometry

**Figure 11:**
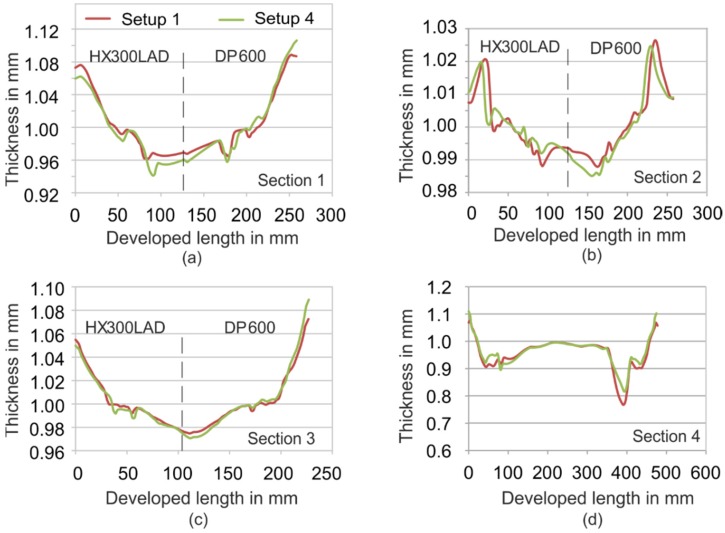
Thickness distribution for Setups 1 (steel/steel) and 4 (steel/polymer K3). (**a**) Section 1; (**b**) Section 2; (**c**) Section 3; (**d**) Section 4.

Since the combination of polymer K3 shows the best results among others, further analyses were carried out for a complex geometry with setups consisting of K3. A constant blank holder force of 100 kN was used for these analyses. Seven springs were used on the polymer side of the die in Setup 6. This led to an extra localized tensile force of 15 kN per spring. Figure 11 shows the thickness distribution on selected sections at the end of the deep drawing process for Setups 1 and 4. It can be seen that the tool with the polymer half (Setup 4) generated results comparable to those of Setup 1 (steel/steel). A relatively large drawing depth of 60 mm was selected in this case to show the application for complex parts. The highest strain occurred in all cases on the side of DP600 at flange. At Section 1, a difference of about 2% can be seen for HX300LAD. The thickness reduction in Setup 4 was smaller than in Setup 1. For Section 2, however, the difference decreased and a better thickness distribution was achieved. For Section 4, at the critical region, the hybrid tool led to about a 4% smaller thickness reduction, as compared to the total steel tool. This validates the idea of the application of tailored tools for the deep drawing of tailored blanks. 

**Figure 12:**
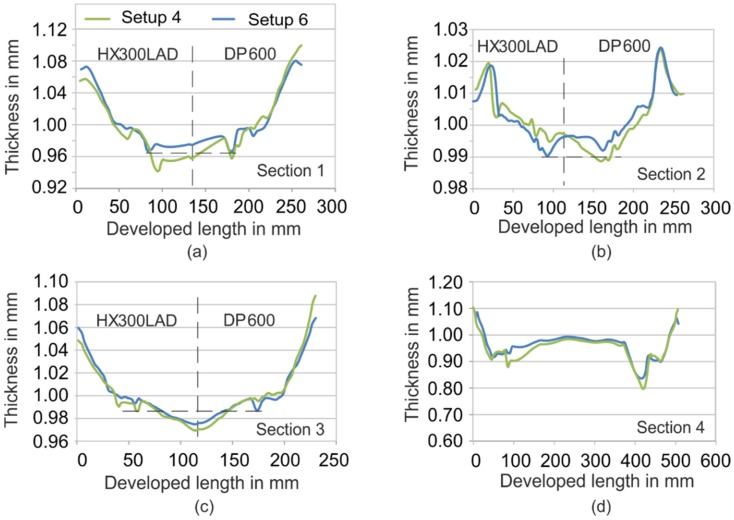
Thickness distribution for setups 4 (steel/polymer K3) and 6 (steel/multi-spring). (**a**) Section 1; (**b**) Section 2; (**c**) Section 3; (**d**) Section 4.

For further analysis, Setup 4 (steel/Polymer K3) and Setup 6 (steel/multi-spring) were simulated, and the results of thickness distribution for 4 sections are shown in Figure 12. It can be seen that at all these sections the hybrid tool with springs shows a smaller thickness reduction. 

**Figure 13:**
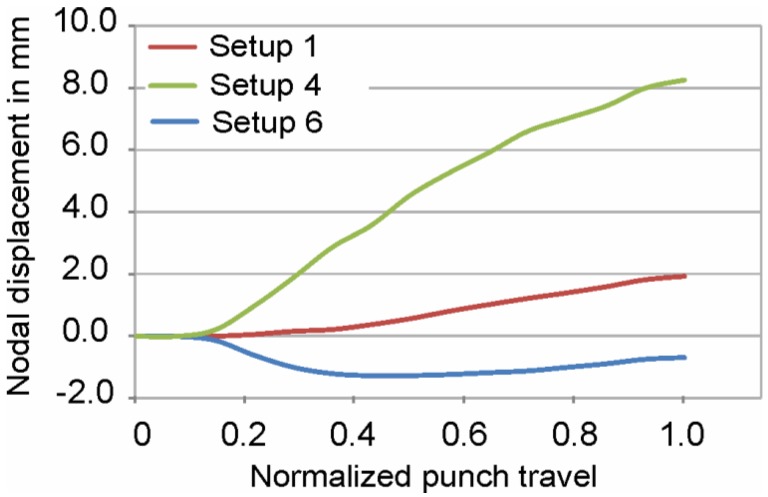
Weld-line movement using nodal displacement for Setups 1 (steel/steel), 4 (steel/polymer K3), and 6 (steel/multi-spring).

Another important result is that the thickness distribution tended to be more uniform with the application of springs. The main reason for this is the control of material flow. This can be further discussed based on the results of weld-line movement, as shown in Figure 13. 

In this case, the nodal displacement was used as a tool to model the weld-line movement over the process time shown here as normalized punch travel. The selected node lay on the intersection of Section 4 and Section 2 (Figure 3c) and, therefore, exactly in the middle of the weld line, experiencing the highest displacement. It can be seen that, although Setup 4 led to a better thickness distribution, the weld-line movement was quite high. The sheet with HX300LAD material deformed more than the side with DP600 under the same loading step. This led to an increased material flow from the HX300LAD side and caused the weld line to move. For the higher blank holder force, the polymer side of Setup 4 tended to deform elastically and therefore generated more clearance and eased the material flow, leading to higher weld-line movement. In order to prevent this effect, the springs were installed, and it can be seen that the weld-line movement for Setup 6 was 0.8 mm, as compared to 2 mm for Setup 1 and 8.2 mm for Setup 4. Another aspect of the weld-line movement for Setup 6 is its variation over the punch travel. The nodal displacement for Setup 6 developed in the negative direction, which means that the material from the DP600 side flowed more than the material from the HX300LAD side. This can be attributed to the strong effect of the springs, which generate localized tension along with the tension generated due to the blank holder force. The nodal displacement increased at the beginning until 40% and then stayed constant for the next 20% of the punch travel. The initial increase is related to the start of the forming operation and shows the higher flow of the DP600 material. After 60% of the punch travel, the hardening effect of DP600 tended to hinder the material flow from the DP600 side and counter the tension effect of springs. This led to an increase in material flow from the HX300LAD side, which can be seen in the last 20% of the punch travel. In this way, by a systematic analysis of the effect of the spring force, a proper requirement of total springs force can be determined and the weld-line movement can be further optimized.

## 4. Conclusions

These investigations on the forming of tailored blanks with tailored tools reveal the possibility of improving the forming behavior of tailored blanks by applying the novel method of using hybrid and steel dies at the same time for different material strengths. The experiments show a lot of positive effects such as the reduction of weld-line movement (Figure 4 and Figure 13), the homogenization of strain (Figure 7a,c,d and Figure 11a,c) and thickness during the forming process, and the weight reduction of tools for press shops. Due to the increase of contact area as a result of deformation of the polymer tools, the material flow of the softer material was hindered successfully. Furthermore, it could be shown that the simulations could be validated with high accuracy for a cylindrical cup and that the positive effects can be transferred to more complex geometries. In the forming process of tailored blanks with hybrid tools, different aspects need to be considered:

- Anisotropic behavior
- Tolerance of thickness in initial blank (0.98 to 1.01 mm)
- Imperfections in polymer tools due to difficulties in the reproducibility of the casting process

Due to the elastic deformation of the polymer die, the geometrical accuracy of the formed cups differed from the original geometry (−0.29 mm); however, the use of polymer for one half of the die can lead to improved formability due to the effect that pressure peaks are avoided with elastic tools. The deviation in geometry of the formed part can be reduced if the elastic deformation is considered in the design process of the tools, and the tools have a larger initial geometry that is compensated by the applied load. Although the density of the polymer is one fourth of the density of the steel, the energy saved in these experiments is negligible as a result of the weight of the ram in the press of more than 300 kg, which is much more than the weight of the used tools. However, for tools with a higher weight, a weight reduction of 75% can lead to lower energy consumption. 
